# Evaluating Long-Term Outcomes in STEMI Patients with New Left Bundle Branch Block: The Impact of Modifiable Risk Factors

**DOI:** 10.3390/jpm14070771

**Published:** 2024-07-19

**Authors:** Larisa Anghel, Bogdan-Sorin Tudurachi, Andreea Tudurachi, Laura-Cătălina Benchea, Alexandra Clement, Răzvan-Liviu Zanfirescu, Radu Andy Sascău, Cristian Stătescu

**Affiliations:** 1 Internal Medicine Department, “Grigore T. Popa” University of Medicine and Pharmacy, 700503 Iași, Romania; bogdan-sorin.tudurachi@d.umfiasi.ro (B.-S.T.); benchea.laura-catalina@d.umfiasi.ro (L.-C.B.); mihaela-alexandra-clement@email.umfiasi.ro (A.C.); radu.sascau@umfiasi.ro (R.A.S.); cristian.statescu@umfiasi.ro (C.S.); 2 Cardiology Department, Cardiovascular Diseases Institute “Prof. Dr. George I. M. Georgescu”, 700503 Iași, Romania; leonteandreea32@gmail.com (A.T.); zanfirescu_razvan-liviu@d.umfiasi.ro (R.-L.Z.); 3 Physiology Department, “Grigore T. Popa” University of Medicine and Pharmacy, 700503 Iași, Romania

**Keywords:** acute myocardial infarction, left bundle branch block, hypertension, diabetes mellitus, dyslipidemia, smoking, long-term follow-up

## Abstract

**Background/Objectives**: Coronary artery disease, a leading global cause of death, highlights the essential need for early detection and management of modifiable cardiovascular risk factors to prevent further coronary events. **Methods**: This study, conducted at a major tertiary academic PCI-capable hospital in Romania from 1 January 2011 to 31 December 2013, prospectively analyzed 387 myocardial infarction with ST-segment elevation (STEMI) patients to assess the long-term management of modifiable risk factors. This study particularly focused on patients with new-onset left bundle branch block (LBBB) and compared them with a matched control group without LBBB. **Results**: During median follow-up periods of 9.6 years for LBBB patients and 9.2 years for those without LBBB, it was found that smoking, obesity, and dyslipidemia were prevalent in 73.80%, 71.42%, and 71.42% of the LBBB group, respectively, at baseline. Significant reductions in smoking were observed in both groups, with the LBBB group’s smoking rates decreasing significantly to 61.90% (*p* = 0.034). Patients with LBBB more frequently achieved low-density lipoprotein cholesterol (LDLc) target levels during the follow-up period (from 71.42% to 59.52%; *p* = 0.026) compared to the control group (from 66.67% to 71.42%; *p* = 0.046). Prescription rates for dual antiplatelet therapy (DAPT), angiotensin-converting enzyme inhibitors (ACEi) or angiotensin II receptor blockers (ARBs), beta-blockers, and statins were initially high but then decreased by the follow-up. Statin use was reduced from 97.62% to 69.04% (*p* = 0.036) in the LBBB group and from 100% to 61.90% (*p* = 0.028) in the non-LBBB group. This study also highlighted moderate correlations between obesity (*r* = 0.627, *p* = 0.040) and subsequent coronary reperfusion in the LBBB group, while dyslipidemia and smoking showed very strong positive correlations across both groups (dyslipidemia: *r* = 0.903, *p* = 0.019 for LBBB; *r* = 0.503, *p* = 0.048 for non-LBBB; smoking: *r* = 0.888, *p* = 0.035 for LBBB; *r* = 0.517, *p* = 0.010 for non-LBBB). **Conclusions**: These findings underscore the crucial need for targeted management of modifiable risk factors, particularly focusing on dyslipidemia and smoking cessation, to improve subsequent coronary reperfusion outcomes post-STEMI, especially in patients with complicating factors like LBBB.

## 1. Introduction

Coronary artery disease remains one of the leading causes of death worldwide [1]. The management of cardiovascular risk factors plays a pivotal role in averting adverse cardiovascular events, such as stroke and ST-segment elevation myocardial infarction (STEMI) [2]. Therefore, early detection and intervention targeting standard modifiable risk factors (SMuRFs), including hypertension, diabetes mellitus, dyslipidemia, and smoking, are imperative for reducing the risk of atherosclerotic cardiovascular disease among all individuals and for preventing cardiovascular events. Recent research indicates an increasing proportion of patients without SMuRFs, termed SMuRF-less, who were previously asymptomatic but now present with ST-segment elevation myocardial infarction [3,4]. The prevalence of patients with STEMI and those who are SMuRF-less among STEMI patients has surged over the past decade from 13% to approximately 27% [5,6]. Strategies that focus on preventing these modifiable risk factors for coronary heart disease are essential. They have not only contributed to significant improvements in treatment but have also led to a substantial reduction in both morbidity and mortality rates [1,7,8].

While recent data indicate a rise in the proportion of patients without SMuRFs, the vast majority of patients with myocardial infarction have at least one modifiable risk factor, and their clinical features and outcomes following hospitalization for STEMI have not been extensively explored [9,10]. Also, changes in the length and pattern of the QRS complex are thought to indicate more acute ischemia and a faster rate of myocardial necrosis development compared to changes in the ST segment alone. There is a continuing debate surrounding whether the presence of new-onset left bundle branch block (LBBB) should be regarded as an independent factor in forecasting the long-term outcomes of patients with STEMI who undergo primary percutaneous coronary intervention (PCI) [11]. This study seeks to evaluate the long-term control of modifiable risk factors in patients with STEMI and new left bundle branch block for devising optimal strategies to manage STEMI risk in this subgroup. Also, we assessed the relationship between managing modifiable cardiovascular risk factors and the occurrence of long-term subsequent coronary reperfusion in patients with STEMI, both with and without new LBBB.

## 2. Materials and Methods

### 2.1. Patient Recruitment and Study Design

Patients presenting with acute myocardial infarction to a major tertiary academic PCI-capable hospital in Romania between 1 January 2011 and 31 December 2013 were prospectively studied. This is the only hospital from the eastern part of Romania that provides PCI services, which include round-the-clock primary PCI. Patients presenting with STEMI arrived either through the Emergency Department of the hospital or via interhospital transfer from the territorial hospitals. The eligible participants in this study were adults aged 18 years and above who presented with STEMI and a single atherosclerotic coronary lesion. Patients who had more than one coronary lesion were excluded from this study to ensure uniformity and concentrate on a specific subset of STEMI patients presenting with a single atherosclerotic coronary lesion. This exclusion was intended to minimize diversity in clinical presentations and treatment outcomes, facilitating a more precise analysis and comparison between patients who developed new-onset LBBB and those who did not. Patients diagnosed with type 2 myocardial infarction, characterized by evidence of myocardial infarction due to an imbalance between myocardial oxygen supply and demand unrelated to acute coronary atherothrombosis, were also excluded. Individuals with a history of previous STEMI, PCI, or coronary artery bypass graft (CABG) were also excluded. Over a period of three years, between 1 January 2011 and 31 December 2013, a total of 387 patients were diagnosed with acute myocardial infarction and a single coronary lesion. Among them, 42 patients were identified to have newly diagnosed left bundle branch block. For comparative purposes, a control group of 42 patients without LBBB was selected through random sampling to ensure representation and comparability with the group of patients with new left bundle branch block. Newly developed left bundle branch block was identified as the onset of LBBB in patients who had not previously experienced this conduction abnormality, and it persisted until the patient was discharged [11]. All participants underwent a prospective evaluation after a median follow-up period of 9.6 years for patients with LBBB and 9.2 years for patients without LBBB. Throughout this follow-up period, various biological parameters were assessed to investigate the impact of an acute coronary event on lifestyle adjustments as well as to evaluate the significance of new LBBB for long-term prognosis in STEMI patients and a single coronary lesion who underwent primary PCI. Modifiable cardiovascular risk factors, referred to as SMuRFs, were defined as having at least one of the following: former or current smoking status, hypertension, diabetes mellitus, or dyslipidemia. Hypertension was characterized by a consistent blood pressure measurement of ≥140/≥90 mmHg [12]. This definition includes patients with a prior diagnosis of hypertension, those prescribed antihypertensive medications, or those newly diagnosed with hypertension during the index admission. Diabetes mellitus was identified as previously diagnosed type 1 or type 2 diabetes, individuals prescribed glucose-lowering medications, or those newly diagnosed with diabetes based on HbA1c levels during the index admission [13]. Dyslipidemia was defined as a previously diagnosed condition, patients prescribed lipid-lowering therapy, or those newly diagnosed with hypercholesterolemia during the index admission [14].

Upon admission, all patients included in this study underwent emergency coronary angiography and received treatment following standard protocols for second-generation drug-eluting stent placement, conducted by experienced and senior cardiologists. After the PCI, all patients received guideline-directed standard treatment regimens, which included contemporary antiplatelet therapy and standard-intensity statin therapy. Regular follow-up appointments were scheduled at the clinic after their discharge.

This study’s average follow-up duration lasted 9.6 years for patients with LBBB and 9.2 years for patients without LBBB and involved clinic visits, the last follow-up visit being at the end of December 2023. At the end of the follow-up period, all 84 patients completed this study, with no patients lost to follow-up. The primary focus of this study was on the long-term control of modifiable risk factors in patients with STEMI and new left bundle branch block to identify what risk factors are associated with poor outcomes and devise optimal strategies to manage STEMI risk in this subgroup. Another objective was to assess the relationship between the control of modifiable cardiovascular risk factors and the occurrence of long-term subsequent coronary reperfusion in patients with STEMI, both with and without new LBBB.

This study was conducted in adherence to the principles outlined in the Declaration of Helsinki and its subsequent amendments, as mandated by the hospital’s Ethical Committee. All data were gathered anonymously, and the patients routinely provided informed consent for the use of their personal data at the onset of their hospitalization. This approach ensured ethical compliance and protected patient confidentiality throughout this study.

### 2.2. Statistical Analysis

Categorical variables were represented by the total count and proportion. Disparities in risk factor management among different groups were assessed using the chi-squared test for categorical variables. Continuous variables with a normal distribution were presented as the mean ± standard deviation. Odds ratios (ORs) along with 95% confidence intervals (CIs) for associations were calculated using multivariable logistic regression. All *p* values reported were two-sided. Statistically significant differences were defined at a significance level of *p* ≤ 0.05. Statistical analysis was conducted utilizing IBM SPSS Statistics version 26.0 (IBM Corp, Armonk, NY, USA).

## 3. Results

Between 1 January 2011 and 31 December 2013, 387 patients with acute myocardial infarction and a single atherosclerotic coronary lesion were admitted to the Intensive Cardiac Care Unit of “Prof. Dr. George I.M. Georgescu” Cardiovascular Disease Institute Iasi. Among them, 42 patients were found to have newly developed left bundle branch block. They were compared with 42 matched control patients without left bundle branch block, treated in the same period. Each participant underwent a prospective evaluation after a median follow-up period of 9.6 years for patients with LBBB and 9.2 years for patients without LBBB.

### 3.1. Baseline Characteristics

The mean age of the patients included in this study was 67 years (52–83) for those with LBBB and 66 years (40–81) for those without LBBB, with a predominance of male gender, 61.9% and 66.66%, respectively. The most frequent symptoms at admission were chest pain and dyspnea. Palpitations and syncope were more frequent in patients without LBBB, but the differences were not statistically significant. Heart rate and systolic blood pressure were also higher in patients without LBBB. Coronary angiography with percutaneous coronary angioplasty of the culprit lesion was performed in all patients included in this study. Regarding the coronary lesions, the left anterior descending artery was responsible for the onset of myocardial infarction in most cases, followed by the circumflex and right coronary arteries in an equal percentage of cases (22.6%) in patients with LBBB and the right coronary artery 26.2% and left circumflex artery 16.7% in patients without LBBB. Overall, while there are variations in lesion percentages between the groups for each artery, none of them are statistically significant (Table 1).

### 3.2. Control of Modifiable Risk Factors

For patients with STEMI and new LBBB, smoking (73.80%) was the most prevalent modifiable risk factor, followed by obesity and dyslipidemia, both in the same percentage, 71.42%. Hypertension (66.66%) and dyslipidemia (66.66%) were the most frequent risk factors for patients without LBBB, followed by obesity (64.28%) and smoking (57.14%). Both groups saw reductions in smoking rates, with the LBBB group showing a significant decrease from 73.80% to 61.90% (*p* = 0.034), while the reduction in the non-LBBB group (from 57.14% to 47.62%) did not reach statistical significance (*p* = 0.068). Dyslipidemia decreased significantly in the LBBB group (from 71.42% to 59.52%; *p* = 0.026) and increased in the non-LBBB group (from 66.67% to 71.42%; *p* = 0.046). This may indicate differing effectiveness in lipid management strategies between the two groups, considering that, at the follow-up visit, only 61.90% of patients without LBBB received lipid-lowering drugs.

The LBBB group showed significant changes in obesity distribution, particularly an increase in higher BMI categories (>35 kg/m^2^) and a decrease in the 30–34.9 kg/m^2^ category, suggesting a redistribution of weight classes (*p* = 0.040). The non-LBBB group also showed changes in BMI distribution, but these were not statistically significant (*p* = 0.088).

A notable finding was the significant reduction in hypertension among the LBBB group, decreasing from 69.04% at baseline to 61.90% at follow-up (*p* = 0.023). In contrast, the non-LBBB group showed an increase from 66.66% to 70.00%, with no significant change (*p* = 0.860). This highlights a potentially more effective management of hypertension in the LBBB group.

Initially, diabetes mellitus was slightly more prevalent in patients with LBBB (28.57%) compared to those without (24.60%). Over the study period, a better control of diabetes was observed in both groups—23.80% in the LBBB group and 19.00% in the non-LBBB group, although these changes did not reach statistical significance (*p* = 0.467 and *p* = 0.520, respectively) (Table 2).

Regarding treatment modifications at discharge and at the long-term follow-up in patients who suffered a STEMI, both with and without new LBBB, significant changes were observed. At baseline, the prescription rates for dual antiplatelet therapy (DAPT), angiotensin-converting enzyme inhibitors (ACEi) or angiotensin II receptor blockers (ARBs), beta-blockers, and statins were notably high in both patient groups.

By the follow-up, DAPT use had decreased dramatically in both groups, falling from 97.62% to 16.66% in the STEMI with LBBB group (*p* = 0.023) and from 97.62% to 23.80% in the STEMI without LBBB group (*p* = 0.029). This significant reduction is not surprising, considering that the guidelines recommend DAPT for at least 12 months after a STEMI.

Treatment with ACEi/ARBs also declined, particularly in the STEMI with LBBB group, from 90.47% to 61.90% (*p* = 0.040). The decrease in the STEMI without LBBB group was not statistically significant, maintaining a level of 61.90% from an initial 78.57% (*p* = 0.560).

Beta-blocker therapy showed stability across the years, with a slight decrease in the STEMI with LBBB group from 92.85% to 85.71% (*p* = 0.467) and a corresponding increase in the STEMI without LBBB group from 78.57% to 85.71% (*p* = 0.238), though these changes were not statistically significant.

Furthermore, there was a notable reduction in statin use at follow-up in both groups. In the STEMI with LBBB group, statin use decreased from 97.62% to 69.04% (*p* = 0.036), and in the STEMI without LBBB group, it dropped from 100% to 61.90% (*p* = 0.028).

These findings highlight significant alterations in the management of STEMI patients over approximately a decade, reflecting changes in clinical guidelines, patient adherence, or other healthcare strategies, particularly in the context of LBBB (Table 3).

### 3.3. Correlations between Control of Modifiable Cardiovascular Risk Factors and the Rate of Long-Term Subsequent Coronary Reperfusion

We also examined the relation between the control of modifiable cardiovascular risk factors and the rate of long-term subsequent coronary reperfusion in patients following STEMI (Table 4).

Dyslipidemia demonstrated very strong correlations with reperfusion rates in both groups (*r* = 0.903, *p* = 0.019 for STEMI with LBBB; *r* = 0.503, *p* = 0.048 for STEMI without LBBB). These results underline the critical impact of lipid control on subsequent coronary reperfusion, indicating that effective management of dyslipidemia could significantly reduce the need for another coronary reperfusion.

Active smoking also showed very strong correlations in both groups (*r* = 0.888, *p* = 0.035 for STEMI with LBBB; *r* = 0.517, *p* = 0.010 for STEMI without LBBB), suggesting that smoking cessation is crucially linked with better long-term outcomes. The strong statistical significance of these relationships points to active smoking as a modifiable factor with a substantial impact on subsequent coronary reperfusion.

Obesity showed a strong correlation with long-term reperfusion rates in the STEMI with LBBB group (*r* = 0.627, *p* = 0.040) but was not significantly correlated in the STEMI without LBBB group. This suggests that, in patients with LBBB, obesity control might play a significant role in the need for subsequent coronary reperfusion strategies.

Diabetes mellitus showed weak correlations with long-term subsequent coronary reperfusion rates in both groups (*r* = 0.123, *p* = 0.780 for STEMI with LBBB; *r* = 0.228, *p* = 0.146 for STEMI without LBBB), suggesting that diabetes control might not have a direct or significant influence on reperfusion outcomes in these groups.

Hypertension displayed a weak correlation in the STEMI with LBBB group, indicating a minimal relationship. However, a moderate positive correlation was observed in the STEMI without LBBB group (*r* = 0.307, *p* = 0.048), indicating a statistically significant association, where better control of hypertension could be related with less need for coronary reperfusion.

These findings suggest that control of certain modifiable risk factors, particularly dyslipidemia and smoking, may significantly influence the rate of subsequent coronary reperfusion post-STEMI. This highlights the importance of comprehensive risk factor management as part of post-STEMI care, especially in patients with complicating factors such as LBBB.

## 4. Discussion

In this study, we analyzed the association between the management of modifiable cardiovascular risk factors and the frequency of long-term subsequent coronary reperfusion in patients with STEMI, with and without new LBBB. Our results indicate that, although factors such as diabetes and hypertension may not directly influence reperfusion requirements, others like obesity, dyslipidemia, and smoking exert significant effects, especially in patients with complicating conditions like LBBB. This underscores the critical role of thorough management of risk factors in the post-STEMI treatment regimen. Also, our findings show marked decreases in the use of DAPT, ACEi/ARBs, and statins during long-term follow-up, with significant changes particularly in patients with new LBBB. While these records provide valuable insights, it is essential to recognize that they may not fully reflect current management practices or the latest therapeutic advances. These changes may be due to shifts in clinical guidelines, variations in patient adherence, or other influences on long-term treatment strategies. However, the findings highlight a targeted approach to managing modifiable risk factors to optimize coronary reperfusion outcomes after STEMI, with a particular focus on dyslipidemia and smoking cessation. The statistically significant alterations underscore the influence of complications like LBBB on the adjustments in treatment over time.

This study involved patients with an average age of 67 years for those with LBBB and 66 years for those without, predominantly male. Common symptoms at admission included chest pain and dyspnea, with palpitations and syncope more frequently reported in patients without LBBB, though not significantly so. All participants underwent coronary angiography and percutaneous coronary angioplasty on the culprit lesion, with the left anterior descending artery most often implicated in myocardial infarctions. The involvement of other arteries—circumflex and right coronary—was similar in LBBB patients and varied slightly in those without LBBB, but these differences were not statistically significant. Previous studies have suggested that the appearance of a new bundle branch block may indicate a more extensive myocardial infarction affecting the proximal portions of the cardiac conduction system, or it could be a manifestation of delayed electrical conduction due to significant ventricular myocardial damage [15]. Over time, guidelines have shifted from recommending automatic reperfusion therapy to more nuanced approaches that incorporate ECG analysis, cardiac biomarker testing, and echocardiography to determine the necessity of emergent reperfusion therapy. This approach allows for a more accurate and quicker diagnosis, aiding in the appropriate management of suspected STEMI cases in patients with LBBB [16,17].

In patients with STEMI and new LBBB, smoking was the most prevalent modifiable risk factor, followed by obesity and dyslipidemia, with significant reductions in smoking and dyslipidemia observed over time. Significant changes in obesity distribution were noted in the LBBB group, indicating shifts in weight classes, whereas hypertension management appeared more effective in this group compared to those without LBBB. Diabetes control showed improvement in both groups, though these changes were not statistically significant. In this study, the rates of controlling modifiable cardiovascular risk factors were low. Other studies from Europe and China, such as the European studies and DYSIS-China, revealed that only approximately 25.6% and 26.86% of coronary heart disease patients, respectively, achieved the recommended low-density lipoprotein cholesterol (LDLc) targets of less than 1.8 mmol/L. Additionally, blood pressure control was adequate in only 55% of the patients, below the set target [18,19]. Data from the CLARIFY registry also indicated that only 59% of stable coronary artery disease patients with hypertension had controlled BP [20]. Similar trends were also observed in our study, with only approximately 40.48% of patients with LBBB and 28.58% of those without LBBB achieving the LDLc targets. This suggests that patients with LBBB were somewhat more successful at reaching LDLc goals compared to those without LBBB. However, the overall achievement rate for both groups was relatively low, indicating a broader issue with managing cholesterol levels effectively in this patient population. All these findings underscore the urgent need for enhanced measures to manage risk factors effectively as part of secondary prevention strategies in patients with coronary artery disease.

Several contemporary studies underscore the critical role of dyslipidemia management in improving post-STEMI outcomes. For instance, recent advancements in lipid-lowering therapies, such as PCSK9 inhibitors, have shown significant benefits in reducing adverse cardiovascular events. A prespecified analysis of the FOURIER trial, involving 5711 patients who had a myocardial infarction within the past 12 months, showed that evolocumab reduced the incidence of the primary endpoint (including cardiovascular death, acute myocardial infarction, stroke, coronary revascularization, or hospitalization for unstable angina) by 19% and the key secondary endpoint (cardiovascular death, acute myocardial infarction, or stroke) by 25%. The study demonstrated that patients who had a recent myocardial infarction experienced a more significant reduction in event risk over a three-year follow-up compared to those whose myocardial infarction occurred more than 12 months prior to randomization. These outcomes confirm that earlier reduction in LDL-C levels results in greater clinical benefits [21].

Our results also indicate significant reductions in the use of DAPT, ACEi/ARBs, and statins at long-term follow-up, particularly notable in patients with new LBBB. The use of beta-blockers remained relatively stable in both groups. The statistical significance of these changes highlights the impact of STEMI complications such as LBBB on long-term treatment modifications. This significant reduction is not surprising, considering that the guidelines recommend DAPT for at least 12 months after a STEMI. This duration is based on evidence showing that the benefits in reducing cardiac risk significantly outweigh the increased risk of bleeding during this period [11].

After the initial year, the continuation of DAPT is considered on a case-by-case basis, extending up to three years or more, particularly for patients at a higher risk of cardiac events but lower risk of bleeding. The decision to extend DAPT beyond the first year involves a careful evaluation of the trade-off between reducing thrombotic risk and increasing bleeding risk. Emerging research suggests potential benefits of personalized DAPT strategies based on genetic markers, platelet reactivity tests, and other biomarkers that could better predict individual risks and benefits. Studies like the DAPT study and others have provided evidence that supports longer DAPT duration in patients with high thrombotic risk but have also highlighted the increased risk of major bleeding [22,23,24]. Ongoing research and patient-specific risk assessments are essential to optimize therapy duration for individual patients, ensuring the maximal benefit with minimal risks.

The observed decrease in the use of ACEi/ARBs among patients who experienced a STEMI with and without new LBBB can be interpreted through several clinical and pharmacological perspectives. Initially, a high usage of ACEi/ARBs is expected in patients following a STEMI because these medications are standard in managing heart failure (HF) and in reducing cardiovascular risks such as hypertension. They are beneficial in improving survival, reducing myocardial stress, and preventing adverse remodeling of the heart after a STEMI [25,26,27,28,29]. The significant decrease from 90.47% to 61.90% in this group might be explained by a reevaluation of cardiovascular risk based on the patient’s current cardiac function, blood pressure control, and the presence of other conditions that might either increase the risk of adverse effects or reduce the necessity for these medications. Also, new clinical guidelines or emerging evidence might have influenced the practice patterns, recommending more personalized approaches or the use of alternative therapies, such as angiotensin receptor-neprilysin inhibitor (ARNI), which have shown a significant benefit in patients with HF [30,31]. The non-significant decrease from 78.57% to 61.90% in patients with STEMI without LBBB suggests a stable but reduced utilization, which could be attributed to similar factors as above but with a less pronounced effect. This stability could also indicate that these patients perhaps did not experience as severe cardiac remodeling or complications as those with LBBB, maintaining a lesser but steady need for ACEi/ARB therapy. Thus, the overall decrease in the usage of ACEi/ARBs in both groups, particularly the statistically significant reduction in the STEMI with LBBB group, suggests a dynSTEMIc approach in the management of post-STEMI patients, emphasizing the importance of regular follow-up and treatment reassessment. Clinicians must weigh the benefits of continued ACEi/ARB therapy against potential risks and changing clinical conditions over time.

The changes observed in beta-blocker therapy among STEMI patients with and without new LBBB reflect a generally stable pattern of medication adherence and clinical decision-making over the long-term follow-up period. The decrease from 92.85% to 85.71% in patients with LBBB might seem notable but is not statistically significant (*p* = 0.467), suggesting that, while some patients discontinued beta-blockers, the overall majority continued their therapy. It is interesting that, in patients with STEMI without LBBB, there was an increase in beta-blocker usage from 78.57% to 85.71%, possibly in response to other underlying risk factors over time. Overall, the changes in beta-blocker usage reflect a balanced approach to post-STEMI care, emphasizing stable and sustained treatment, where benefits are maximized and risks are carefully managed based on the evolving clinical status of each patient. In a recent study, Lee et al. explored the impact of prolonged beta-blocker therapy post-MI, using data from a nationwide Korean registry of patients treated between 2004 and 2014. Their analysis, which included 7159 patients who received PCI and remained event-free for three months post-MI, demonstrated that longer beta-blocker usage significantly reduced the risks of all-cause death, recurrent MI, and HF over an average follow-up period of 5 years. Their study provides valuable insights into the benefits of extended beta-blocker therapy beyond the typical duration recommended in the clinical guidelines [32]. In contrast, the REDUCE-STEMI trial conducted across Sweden, Estonia, and New Zealand, which used a more contemporary patient population with preserved left ventricular function and modern MI treatments, found no significant benefit from long-term beta-blocker use [33]. These contrasting findings highlight the evolving understanding of beta-blocker efficacy in different MI patient subsets, suggesting that the benefits of long-term beta-blocker use may be more pronounced in certain historical or higher-risk populations.

The significant reductions in statin use among patients post-STEMI, both with LBBB (from 97.62% to 69.04%; *p* = 0.036) and without LBBB (from 100% to 61.90%; *p* = 0.028), raises concerns about the long-term risk management of coronary artery disease. Reduction in statin use at follow-up could be influenced by some side-effects or patient compliance issues. Statins not only help lower cholesterol levels but also have pleiotropic effects that include improving endothelial function, reducing inflammation, and stabilizing atherosclerotic plaques [34]. Reduction in their use might impact the long-term prognosis, especially in these very high-risk populations. This underscores the need for personalized, patient-centered approaches, ensuring patient adherence, managing side-effects, and continuously reassessing treatment necessity in order to optimize the long-term outcomes.

The correlations between the control of modifiable cardiovascular risk factors and the rate of long-term subsequent coronary reperfusion provide significant insights into potential intervention points for improving patient outcomes after a STEMI. Although overall control of modifiable cardiovascular risk factors was found to be low, the rates of no smoking and LDLc control in patients with recurrent coronary events were lower than those in patients without subsequent coronary reperfusion needs. The very strong correlations observed for dyslipidemia (*r* = 0.903, *p* = 0.019 for STEMI with LBBB; *r* = 0.503, *p* = 0.048 for STEMI without LBBB) underline dyslipidemia as a critical modifiable risk factor with a profound impact on subsequent coronary reperfusion needs. Effective lipid management could significantly reduce the progression of coronary artery disease, thereby potentially diminishing the necessity for further reperfusion procedures, reflecting its pivotal role in maintaining vascular health post-STEMI [14,35,36]. A recent retrospective study evaluated the adequacy of lipid management in post-MI patients as compared to the 2019 European Society of Cardiology Guidelines for the management of dyslipidemia. Only 14.7% of the patients studied achieved the guideline-recommended LDL-C goal of an LDL-C target of <1.4 mmol/L and a ≥ 50% reduction from baseline LDL-C after follow-up, underscoring the need for enhanced secondary prevention measures in line with current guidelines [37].

Similarly, active smoking demonstrated very strong correlations in both patient groups (*r* = 0.888, *p* = 0.035 for STEMI with LBBB; *r* = 0.517, *p* = 0.010 for STEMI without LBBB). These results emphasize the substantial negative impact of smoking on cardiovascular health and its strong association with the need for subsequent coronary reperfusion interventions. Moreover, the importance of smoking cessation has been reiterated in numerous studies, highlighting its substantial impact on reducing mortality and improving long-term outcomes in STEMI patients. A recent study explored changes in smoking habits among 322 patients who experienced an acute myocardial infarction while being smokers. Approximately 48.2% of these patients successfully quit smoking after their heart attack, with a significant influence noted from doctor’s advice, indicating a five-fold increase in the likelihood of quitting. Factors such as nicotine withdrawal and the visual impact of health warnings on cigarette packs also played substantial roles in smoking cessation efforts among these patients [38]. Considering the important role of smoking in increasing the risk of mortality after a STEMI, a recent trial from the United States is evaluating an integrated treatment approach for these patients. This approach combines smoking cessation and mood management over 12 weeks, comparing it with a control group receiving general health education and smoking cessation support. The efficacy of this treatment is being assessed through follow-up evaluations up to 12 months post-hospital discharge, with further tracking of cardiac events and mortality for 36 months. The study aims to provide insights into the effectiveness of addressing both smoking and mood in improving post-STEMI outcomes [39]. In a study of 765 patients aged ≤60 years, continued smoking one year after STEMI was associated with a higher risk of major adverse cardiovascular events and death compared to nonsmokers, whereas those who quit smoking had long-term outcomes comparable to nonsmokers. These findings emphasize the substantial health benefits of smoking cessation post-STEMI despite the low cessation rates observed in practice [40]. Our findings align with these studies, emphasizing the need for robust smoking cessation programs as an integral part of post-STEMI care. Smoking cessation appears to be a crucial intervention that can markedly improve long-term outcomes.

Also, recent studies demonstrated significant associations between mixed metal exposure and increased systemic atherosclerotic burden, primarily driven by cadmium (Cd), copper (Cu), and lead (Pb) [41,42,43,44]. Specifically, arsenic (As) and Cu were major contributors to coronary atherosclerosis, while Cd, Cu, and Pb significantly influenced carotid and lower-limb atherosclerosis. Notably, interactions between Cd and Pb or chromium (Cr) were observed, along with interactions of Pb with age, sex, and alcohol consumption. The study concluded that, in CAD patients, exposure to a combination of metals is significantly associated with increased systemic atherosclerotic burden, with pronounced effects in peripheral and carotid arteries [45]. Smoking is the primary source of cadmium exposure for individuals not exposed to industrial environments and also a notable source of lead exposure. These findings highlight the importance of controlling environmental metal exposure to reduce systemic atherosclerosis in CAD patients.

The strong correlation observed for obesity in the STEMI with LBBB group (*r* = 0.627, *p* = 0.040) but not in the STEMI without LBBB group underscores the importance of obesity control in specific subgroups of STEMI patients. For those with LBBB, obesity may significantly exacerbate the need for subsequent coronary reperfusion strategies, likely due to the additional cardiovascular stress imposed by excessive body weight. In a comprehensive Swedish cohort study (*n* = 1,668,921; mean age, 18.3 years; 1968–2005), researchers explored the link between BMI and the risk of early acute coronary events during a follow-up period of up to 48 years. The study revealed that individuals with a BMI of 35 kg/m^2^ had a hazard ratio (HR) of 4.84 for experiencing such events before the age of 40, even after adjusting for fitness and cognitive measures. This finding underscores the growing concern over the obesity epidemic and its potential to increase the coronary heart disease incidence, especially given the rising rates of overweight and obesity among young adults [46].

The impact of hypertension control shows varied significance based on the presence of LBBB. In patients with LBBB, there is only a weak correlation, indicating minimal direct impact on reperfusion rates. Conversely, in patients without LBBB, a moderate positive correlation (*r* = 0.307, *p* = 0.048) suggests that effective hypertension management is significantly associated with a reduced need for subsequent coronary reperfusion. Also, based on our results, diabetes control may not have a significant direct impact on the need for subsequent coronary reperfusion interventions.

Despite the advances in reperfusion strategies, the phenomenon of no-reflow, characterized by inadequate myocardial perfusion despite successful revascularization, remains a significant predictor of poor outcomes. Identifying patients at a higher risk for no-reflow and subsequent major adverse cardiovascular events is crucial for improving clinical outcomes. The SYNTAX II score (SS-II) integrates anatomical and clinical variables to enhance risk stratification in patients undergoing PCI. A recent study examined the relationship between the SYNTAX II score and no-reflow on electrocardiography in predicting in-hospital major adverse cardiovascular events in STEMI patients. Among 126 patients undergoing primary PCI, those with incomplete ST-segment resolution (no-reflow) had significantly higher SS-II scores. Both SS-II and no-reflow were associated with an increased risk of in-hospital major adverse cardiovascular events. Logistic regression analysis confirmed that SS-II and incomplete ST-segment resolution are significant predictors of in-hospital major adverse cardiovascular events [47]. While our study highlights the potential importance of the control of modifiable cardiovascular risk factors and the rate of long-term subsequent coronary reperfusion in patients following STEMI, it is important to acknowledge that we did not evaluate the relationship between the SYNTAX II score and long-term coronary reperfusion. Future research should address this limitation by examining the impact of SS-II and no-reflow on long-term outcomes to provide a more comprehensive understanding of their clinical utility.

There are some limitations to this study. This study’s findings are based on a relatively small cohort from a single tertiary academic hospital in Romania, which may limit the generalizability of the results to other populations or settings. Although this study prospectively analyzed data for a median follow-up period of almost 10 years, it relied on historical patient records from 2011 to 2013, which might not fully capture current management practices or the latest therapeutic advances. There was a significant reduction in the use of statins and other medications over the follow-up period. The reasons for this decline, whether due to non-compliance, side-effects, or changes in clinical guidelines, were not fully explored.

However, there are many strengths of this study that collectively enhance the credibility and utility of this study, providing meaningful contributions to the field of cardiovascular disease management, particularly in patients with complicated presentations like LBBB post-STEMI. This study benefits from prospectively collected data, which likely improves the accuracy and reliability of the information gathered, as opposed to relying solely on retrospective data collection. With up to 9.6 years of follow-up, this study provides valuable long-term insights into the management and outcomes of STEMI patients, particularly those with and without LBBB, which is crucial for understanding the chronic nature of coronary artery disease. This study meticulously assessed a comprehensive set of modifiable risk factors, including smoking, obesity, and dyslipidemia, and their management over time, providing a detailed view of patient profiles and treatment changes. By specifically analyzing patients with new-onset LBBB, this study addresses a subgroup of STEMI patients who are often underrepresented in cardiovascular research, offering targeted insights that could influence future guidelines and interventions.

## 5. Conclusions

Overall, our findings advocate for a targeted approach in managing modifiable risk factors, especially focusing on dyslipidemia and smoking cessation, to optimize subsequent coronary reperfusion outcomes post-STEMI. The significant correlations suggest that, while some factors like diabetes and hypertension might not directly dictate reperfusion needs, others like obesity, dyslipidemia, and smoking have potent impacts, particularly in patients with complicating factors such as LBBB. This emphasizes the importance of comprehensive risk factor management as an integral part of post-STEMI care.

## Figures and Tables

**Table 1:** Overview of baseline demographic and clinical characteristics.

Variable	STEMI with New LBBB (*n* = 42 Patients)	STEMI without LBBB (*n* = 42 Patients)	*p* Value
**Baseline characteristics**			
**Age (years)**	67 (52–83)	66 (40–81)	0.864
**Male**	61.90%	66.66%	0.760
**Urban area**	59.52%	64.28%	0.630
**Symptoms at admission**			
**Chest pain**	97.62%	95.24%	0.876
**Dyspnea**	73.80%	66.66%	0.508
**Syncope**	7.14%	16.66%	0.560
**Palpitations**	2.38%	4.76%	0.895
**Admission hemodynSTEMIcs**			
**Mean SBP (mmHg)**	132	135	NA
**Mean HR (bpm)**	67	70	NA
**Oxygen saturation aa (%)**	92%	94%	0.234
**Coronary lesion**			
**LAD**	54.8%	57.1%	0.780
**LCX**	22.60%	16.7%	0.345
**RCA**	22.60%	26.2%	0.456

STEMI, acute myocardial infarction; HR, heart rate; LAD, left anterior descending artery; LBBB, left bundle branch block; LCX, left circumflex artery; NA, not applicable; RCA, right coronary artery; SBP, systolic blood pressure.

**Table 2:** Control of modifiable risk factors for patients included in this study.

Variable	STEMI with New LBBB (*n* = 42 Patients)			STEMI without LBBB (*n* = 42 Patients)		
	Baseline	Follow-Up (Median 9.6 Years)	*p* Value	Baseline	Follow-Up (Median 9.2 Years)	*p* Value
**Diabetes mellitus**	28.57%	23.80%	0.467	24.60%	19.00%	0.520
**Hypertension**	69.04%	61.90%	**0.023**	66.66%	70.00%	0.860
**Obesity** **BMI (kg/m^2^)**	71.42%	64.25%	**0.040**	64.28%	61.90%	0.088
**25–29.9**	4.76%	9.52%		7.14%	9.52%	
**30–34.9**	54.76%	38.09%		42.85%	45.53%	
**35–39.9**	14.29%	16.66%		19.04%	14.29%	
**>40**	2.38%	9.52%		2.38%	2.38%	
**Dyslipidemia**	71.42%	59.52%	**0.026**	66.66%	71.42%	**0.046**
**Active smoker**	73.80%	61.90%	**0.034**	57.14%	47.62%	0.068

STEMI, acute myocardial infarction; BMI, body mass index; LBBB, left bundle branch block.

**Table 3:** Treatment at discharge and follow-up for patients included in this study.

Variable	STEMI with New LBBB (*n* = 42 Patients)			STEMI without LBBB (*n* = 42 Patients)		
	Baseline	Follow-Up (Median 9.6 Years)	*p* Value	Baseline	Follow-Up (Median 9.2 Years)	*p* Value
**DAPT**	97.62%	16.66%	0.023	97.62%	23.80%	0.029
**ACEi/ARBs**	90.47%	61.90%	**0.040**	78.57%	61.90%	0.560
**Beta-blockers**	92.85%	85.71%	**0.467**	78.57%	85.71%	0.238
**Statins**	97.62%	69.04%	**0.036**	100%	61.90%	**0.028**

ACEi/ARBs, angiotensin-converting enzyme inhibitors/angiotensin II receptor blockers; STEMI, acute myocardial infarction; DAPT, dual antiplatelet therapy; LBBB, left bundle branch block.

**Table 4:** Correlations between control of modifiable cardiovascular risk factors and the rate of long-term subsequent coronary reperfusion.

Variable	STEMI with New LBBB (*n* = 42 Patients)		STEMI without LBBB (*n* = 42 Patients)	
	Pearson r Correlation Coefficient	*p* Value	Pearson r Correlation Coefficient	*p* Value
**Diabetes mellitus**	0.123	0.780	0.228	0.146
**Hypertension**	0.238	0.678	0.307	**0.048**
**Obesity**	0.627	**0.040**	0.194	0.210
**Dyslipidemia**	0.903	**0.019**	0.503	**0.048**
**Active smoker**	0.888	**0.035**	0.517	**0.010**

STEMI, acute myocardial infarction; LBBB, left bundle branch block.

## Data Availability

All data generated or analyzed during this study are included in this article.
